# Limited Effect of Biodiversity on the Multifunctionality of a Revegetated Riparian Ecosystem

**DOI:** 10.3390/microorganisms13030554

**Published:** 2025-02-28

**Authors:** Yueliang Jiang, Chen Ye, Manuel Delgado-Baquerizo, Guiyao Zhou, Yu Gong, Quanfa Zhang

**Affiliations:** 1 POWERCHINA Chengdu Engineering Co., Ltd., Chengdu 610072, China; wiup@163.com; 2 Key Laboratory of Aquatic Botany and Watershed Ecology, Wuhan Botanical Garden of the Chinese Academy of Sciences, Wuhan 430074, China; yechen922@wbgcas.cn (C.Y.); qzhang@wbgcas.cn (Q.Z.); 3 Danjiangkou Wetland Ecosystem Field Scientific Observation and Research Station, The Chinese Academy of Sciences & Hubei Province, Wuhan 430074, China; 4 Laboratorio de Biodiversidad y Funcionamiento Ecosistémico, Instituto de Recursos Naturales y Agrobiología de Sevilla (IRNAS), Consejo Superior de Investigaciones Científicas, Av. Reina Mercedes 10, 41012 Sevilla, Spain; m.delgado.baquerizo@csic.es (M.D.-B.); jdzhouguiyao@163.com (G.Z.); 5 Unidad Asociada CSIC-UPO (BioFun), Universidad Pablo de Olavide, 41013 Sevilla, Spain; 6 German Centre for Integrative Biodiversity Research (iDiv) Halle-Jena-Leipzig, Puschstrasse 4, 04103 Leipzig, Germany

**Keywords:** ecosystem multifunctionality, natural rewilding, revegetation, riparian ecosystem

## Abstract

Vegetation and microbial diversity play an essential role in ecosystem function. Active ecosystem restoration costs millions of dollars to increase biodiversity, yet when and how this restoration is effective when aiming at restoring multiple ecosystem functions (EMF) is still under debate. Here, we investigated the influence of a decade of restoration practices (i.e., active revegetation vs. natural rewilding) on the recovery of the ecosystem multifunctionality (EMF) provided by a riparian ecosystem. The experiment was conducted within the region of China’s Three Gorges Dam, and the area was subjected to a gradient of flooding disturbance. We found that active revegetation increased the plant diversity by 13~57% and EMF by ~2.6 times at the extreme flooding zone (~286 flooding days/year) of the riparian ecosystem, when compared with natural rewilding. Moreover, the positive relationship between plant diversity and EMF was weak, and abiotic factors (soil aggregate, pH, soil water content, and heavy metal content) were the dominant predictors for EMF, explaining 52% of the EMF variation. Revegetation impacted EMF both directly and indirectly via altering the soil properties. In addition, we also observed important trade-offs between plant biomass and soil functions (carbon storage and fertility). This study provides critical insights into whether and how a decade of active restoration is effective to recover the EMF supported by riparian ecosystems, and it highlights the importance of active revegetation in conservation and management programs for riparian ecosystems under future extreme flooding conditions.

## 1. Introduction

The United Nations Decade on Restoration is fueling multiple global initiatives to understand how to restore ecosystems across the globe to mitigate climate change and promote nature-based ecosystem services. Active restoration is considered a potent tactic to protect biodiversity, maintain habitat complexity, and enhance human well-being [1,2]. However, its capacity to restore ecosystem functions compared with natural rewilding is poorly understood [3], and it is necessary to consider the contrasting levels of disturbance intensity, as it may only be efficient for a particular level of disturbance. This knowledge is essential in incentivizing governments and institutions to allocate investments towards degraded ecosystem restoration on a global scale, precisely where such restoration is required. Moreover, most studies concentrate on a particular aspect of ecosystem function, such as carbon stocks and productivity [4,5]. The influences of active and natural restoration on multiple ecosystem functions (i.e., ecosystem multifunctionality, EMF) remain less explored.

Here, we used riparian ecosystems as our system model to investigate the capacity of active and natural rewilding in influencing multiple ecosystem functions under contrasting levels of flooding disturbance. Riparian ecosystems, extending from the water’s edge to the uplands, are subjected to severe degradation by anthropogenic activities such as land use change and hydroengineering facilities [6]. Their protection and restoration have been one of the top environmental priorities across the world [7]. Our current understanding of the efficiency of revegetation in supporting multiple ecosystem functions of riparian ecosystems is limited due to the insufficient comprehension of the multiple dimensions of ecosystem functions and a lack of knowledge regarding where active restoration is more efficient than natural rewilding. For example, many restoration studies focus on individual ecosystem functions. Vegetation productivity, carbon stocks, and soil fertility have been demonstrated to increase with revegetation [4,5,8]. EMF is a crucial concept in both ecology and management, providing a robust foundation for the comprehensive integration of multiple ecosystem functions [9]. While caution is necessary during the formulation and comprehension of multifunctionality metrics, this approach is widely recognized as crucial in cultivating a holistic understanding of ecosystem functionality [10]. Quantifying the extent to which ecosystem multifunctionality changes with revegetation restoration and how these effects change across different flooding zones are thus integral in addressing challenges related to the functional recovery of disturbed riparian ecosystems. Moreover, regarding revegetation restoration, it is necessary to explore the potential trade-offs among the ecosystem functions with the goal of promoting long-term sustainable ecosystems [11]. Failure to do so will impede us in better understanding how revegetation might affect ecosystem sustainability and future climate change mitigation in riparian ecosystems.

Revegetation restoration is generally considered to promote EMF via increasing biodiversity [12]. However, the relationship between EMF and biodiversity is mainly gained from theoretical studies and from experiments in which biodiversity is manipulated [13]. Their relationship in real-world situations has not been well studied. In addition, although biodiversity has been widely considered as the dominant driver for ecosystem multifunctionality [14], the relative importance of biodiversity versus soil properties is still under debate. Some studies have demonstrated that soil properties are equally as important as biodiversity in determining the multifunctionality of natural ecosystems [13,15,16]. Whether this is the case in the riparian ecosystem is unknown.

To fill this gap, we took advantage of a decade of revegetation restoration within the riparian zone of the Three Gorges Dam and investigated the response of ecosystem multifunctionality to revegetation restoration at different flooding zones. The Three Gorges Dam is the world’s largest hydroelectric project, spanning 1080 km^2^ [17]. Such a project has changed the hydrological regime and reduced the biodiversity of the ecosystem [18]. To restore and protect riparian ecosystems, active revegetation has been carried out for at least 10 years. Flooding-resistant plants like *Cynodon dactylon*, *Hemathria sibirica*, *Hibiscus syriacus*, *Morus alba*, *Salix variegate*, *Salix chaenomeloides*, and *Taxodium distichum* were selected for this revegetation [19]. Our investigation included four essential ecosystem functions, i.e., plant productivity, microbial habitats, soil carbon stocks, and fertility [20]. The multifunctionality was determined using the averaging multifunctionality index, multiple dimensions of functions, and the multiple-threshold approach [9,21]. We sought to address the following pivotal questions: (1) How does active revegetation affect the EMF of riparian ecosystems? (2) Are there any potential trade-offs among multiple functions during riparian ecosystem restoration? (3) What are the major drivers of EMF during riparian ecosystem restoration?

## 2. Materials and Methods

### 2.1. Site Description

**Figure 1:**
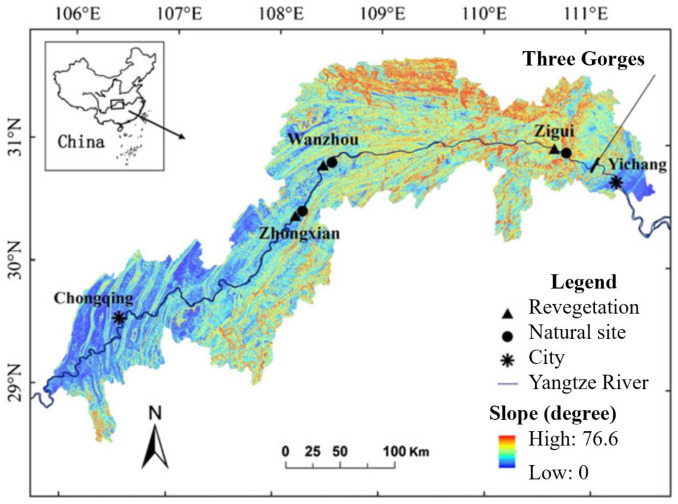
Study site.

This study was conducted at the riparian zone of the Three Gorges Dam (29°16′–31°25′ N, 106–111°50′ E) in China (Figure 1). The riparian area exhibits a humid subtropical monsoon climate. The annual average precipitation in this area ranged from 878 to 1524 mm, and the annual average temperature ranged from 16.3 to 18.2 °C (1971–2001) [22]. The riparian ecosystem can be divided into three distinct zones based on the flooding intensity: the extreme flooding zone (EFZ), the severe flooding zone (SFZ), and the moderate flooding zone (MFZ). They experienced ~286 days, ~237 days, and ~169 days of flooding per year, respectively [19]. Active revegetation has been conducted, accompanying natural rewilding, at three restoration sites (Zhongxian, Wanzhou, and Zigui).

### 2.2. Soil Properties

The soil was collected semi-annually (June and September) from 2013 to 2018, while restoration began in 2010. There were three flooding zones in each sampling site, and one randomly selected plot (1 × 1 m) was established at each flooding zone. Five soil samples were obtained from the plot by a stainless-steel corer (5 cm in diameter and 20 cm in depth). These individual samples were thoroughly mixed to generate a composite sample. The samples were securely enclosed within plastic bags and subsequently maintained at a temperature of 4 °C in preparation for analysis.

The soil moisture content and bulk density were measured by conducting a process that involved weighing fresh soil samples using a known volume ring, followed by oven-drying at 105 °C for a duration of 24 h. The soil pH was measured in a soil-to-water solution with a 2:1 weight ratio using a Fisher Scientific AR15 pH device (Waltham Inc., Waltham, MA, USA). The soil aggregate was determined by the hydrometer method. For the determination of the soil organic matter content (SOM), the K_2_Cr_2_O_7_ titration method was employed following digestion. The analysis of total nitrogen (TN) was conducted using the Kjeldahl method. The extraction of NO_3_^−^-N was carried out using pure water and subsequent measurement was performed utilizing the phenol disulfonic acid spectrophotometric technique. The extraction of NH_4_^+^-N from fresh soil samples was achieved by employing a 2 M KCl solution, and its concentration was determined using indophenol blue colorimetry. The analysis of total phosphorus (TP) was accomplished through molybdenum blue colorimetry, while the available phosphorus (AP) was determined using the colorimetric method. Flame photometry was used to measure the total potassium (TK) and available potassium (AK).

There were nine heavy metals measured in this study. The concentrations of Cr, Cd, Pb, Cu, Zn, Fe, and Mn were determined using flame atomic absorption spectrometry (Analytik Jena AG Inc., Jena, Germany), and the concentration of Hg was determined by cold vapor AAS (Analytik Jena AG Inc., Jena, Germany). The As concentration was measured by the diethyl disulfide and carbamate silver colorimetric method.

### 2.3. Vegetation Diversity and Biomass

In each flooding zone of the sampling sites, four quadrats (1 m × 1 m for herbs, 5 m × 5 m for shrubs, and 10 m × 10 m for trees) were examined. The plant species present within these quadrats were identified, and four plant diversity indices were measured, namely the richness, Shannon–Wiener index (H), Simpson’s index (D), and Alatalo’s evenness index (Ea) [23]. The above-ground biomass of herbs at different flooding zones was determined using the harvesting method.

### 2.4. Microbial Characteristics

The plate counting method was used to measure the abundances of bacteria, fungi, and actinobacteria. Plate counts of culturable bacteria were performed on tryptone soya agar amended with 0.1 g L^−1^ cycloheximide. Plate counts of the colony-forming units (cfu) of fungi were performed on rose Bengal agar amended with 30 mg L^−1^ streptomycin sulfate. Plate counts of the cfu of actinobacteria were performed on glycerol casein agar amended with 0.05 g L^−1^ cycloheximide. Plates were inoculated with 100 μL of soil suspension and incubated at 25 °C for seven days, after which visible colonies of microorganisms were present [24,25]. Control plates, containing the respective media but without any suspension, were also incubated to test potential contamination effects.

The chloroform fumigation–extraction method was used to measure the microbial biomass carbon (MBC) and nitrogen (MBN) [26]. Three sub-samples of each soil, each weighing 5.0 g, underwent fumigation with chloroform devoid of ethanol at 25 °C for a duration of 24 h within an evacuated extractor. The other three sub-samples that had not been fumigated were considered as controls. Subsequently, these soils were subjected to extraction using 20 mL of 0.5 M K_2_SO_4_ solution through horizontal shaking for an hour. The extracts were then filtered and stored at −15 °C prior to undergoing chemical analysis. The Multi N/C 3100 analyzer (Analysensysteme GmbH Inc., Jena, Germany) was utilized to assess the concentrations of total organic carbon and total nitrogen within these extracts. The concentrations of MBC and MBN in the soil were computed using conversion factors of 0.45 and 0.54, respectively.

### 2.5. Ecosystem Multifunctionality

There were four groups of ecosystem functions: soil fertility (i.e., nitrogen, potassium, and phosphorus), soil carbon storage (calculated based on soil organic matter concentration and bulk density), plant biomass, and microbial habitats (i.e., microbial biomass carbon and nitrogen) [20]. Three EMF approaches were utilized in this study: the averaging approach, multidimensional approach and multi-threshold approach. Specifically, the averaging approach provides a clear and easily understandable metric through which to gauge the restorative efficiency in sustaining multiple functions simultaneously. Each variable was normalized and standardized. These standardized variables were averaged to obtain an averaging function index [15,16]. The multiple-threshold approach quantifies the functions that concurrently surpass distinct thresholds (>25%, 50%, 75%, and 90%) of maximum values. It assesses whether a greater (or lesser) number of functions are operating simultaneously at different levels [9]. In addition, principal coordinate analysis (PCoA) was used to determine the multiple dimensions of ecosystem functions [11].

### 2.6. Statistical Analysis

The impacts of the restoration approaches (i.e., active revegetation and natural rewilding) and flooding intensity (i.e., EFZ, SFZ, and MFZ) on the EMF were investigated by a linear mixed-effects model. The plot and year were considered as random factors. The significant differences among the flooding intensities and restoration approaches were analyzed by the post hoc Tukey test. Multiple regression models were applied to investigate the impacts of the soil properties (soil aggregates, pH, SWC, and heavy metals) on EMF. For the soil aggregate and heavy metal content, principal component analysis (PCA) was used for dimensionality reduction. Additionally, structural equation modeling (SEM) was applied to further investigate the direct and indirect effects of the flooding intensity and revegetation on EMF, using the “piecewiseSEM” package in R version 4.3.1. The goodness of the modeling results was assessed by Fisher’s C-test.

## 3. Results

### 3.1. The Effects of Revegetation and Flooding on Ecosystem Multifunctionality

**Figure 2:**
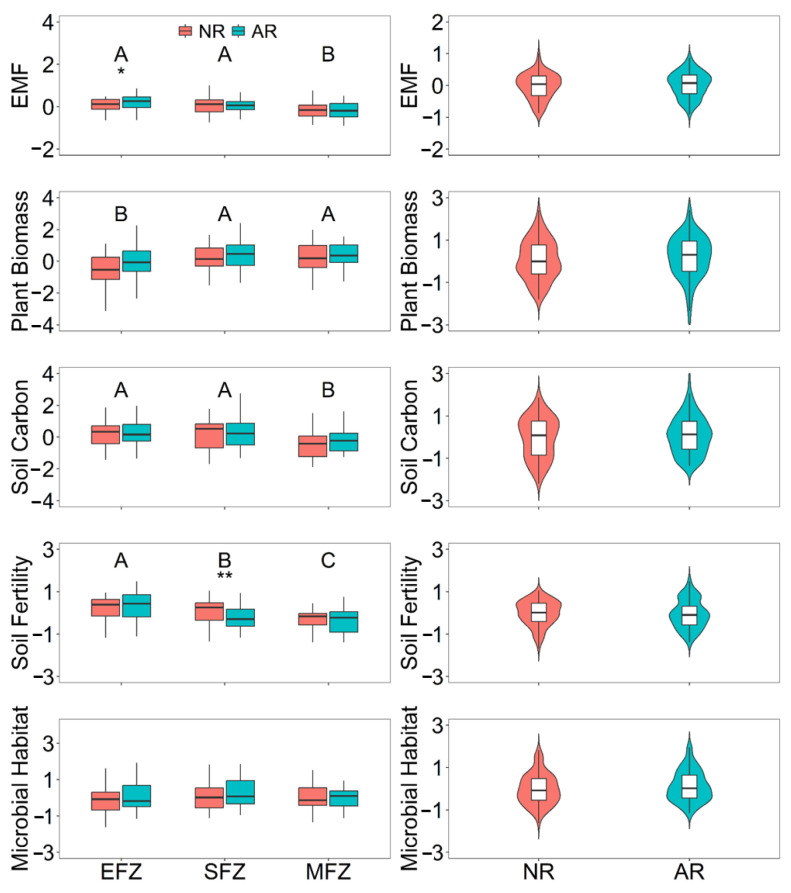
The impact of restoration approaches on the four individual ecosystem functions and total multifunctionality (EMF) under different flooding intensities. “NR” represents natural rewilding, while “AR” represents active revegetation. “EFZ”, “SFZ”, and “MFZ” represent the extreme flooding zone, severe flooding zone, and moderate flooding zone, respectively. The different capital letters represent the significant differences among the flooding intensities. “*” and “**” represent the significant differences between natural rewilding and active revegetation at the level of *p* < 0.05 and *p* < 0.01, respectively.

**Figure 3:**
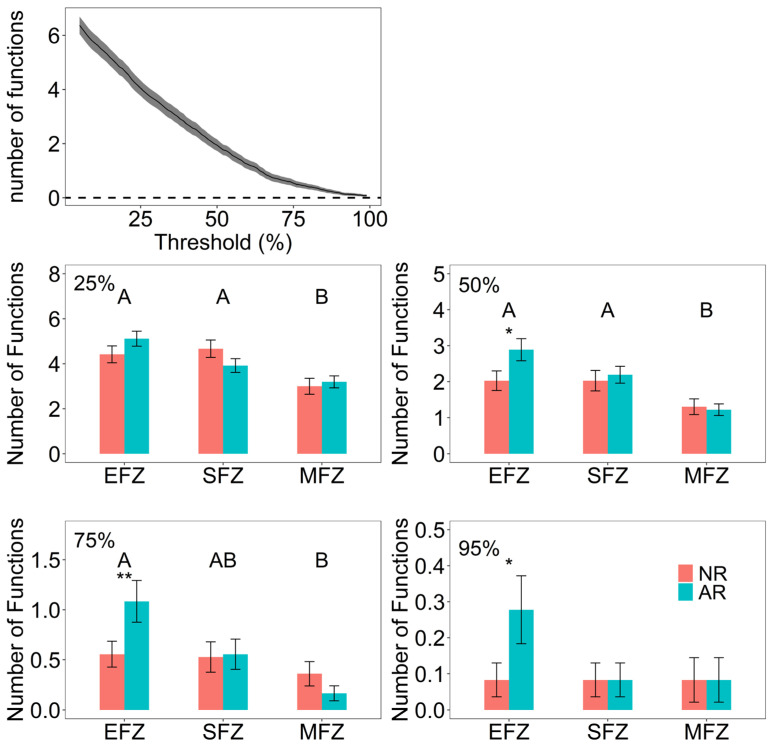
The impact of restoration approaches on the multifunctionality at different flooding zones. The multifunctionality was calculated by the threshold approach at the levels of 25%, 50%, 75%, and 95%. “*” and “**” represent the significant difference between the restoration approaches at the levels of *p* < 0.05 and *p* < 0.01, respectively. The different capital letters represent the significant differences among the flooding intensities. “NR” represents natural rewilding, while “AR” represents active revegetation.

Compared with natural rewilding, active revegetation had significantly higher EMF by 2.6 times at the EFZ (Figure 2), which was also observed when using the multiple-threshold approach (Figure 3). In contrast, this positive effect of active revegetation was not observed at the SFZ and MFZ. In terms of individual functions, active revegetation significantly decreased the soil fertility by 3.5 times at the SFZ compared with natural rewilding (Figure 2).

**Figure 4:**
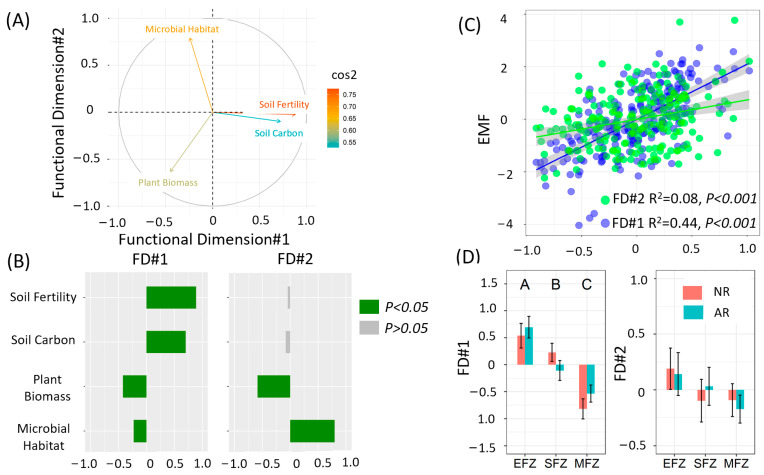
(**A**) Principal coordinate analyses of multiple ecosystem functions. (**B**) The relationship between functional dimensions and functions. (**C**) The relationship between functional dimensions and ecosystem multifunctionality. (**D**) The effects of restoration and hydrological change on the functional dimension. “EFZ”, “SFZ”, and “MFZ” represent the extreme flooding zone, severe flooding zone, and moderate flooding zone, respectively. Different letters represent significant differences between flooding zones.

The PCA results revealed that functional dimensions #1 and #2 explained 69% of the functional variation (Figure 4A). The soil carbon and soil fertility contributed with positive loadings to functional dimension #1 (Figure 4B). Meanwhile, functional dimension #2 was negatively correlated with plant biomass but positively correlated with microbial habitats (Figure 4B). Moreover, EMF was positively correlated with functional dimensions #1 and #2 (Figure 4C). Functional dimension #1 increased with the flooding intensity, while functional dimension #2 was not impacted by the flooding intensity (Figure 4D). Furthermore, revegetation significantly impacted functional dimension #1 at the SFZ (Figure 4D).

### 3.2. The Drivers of Ecosystem Multifunctionality

**Figure 5:**
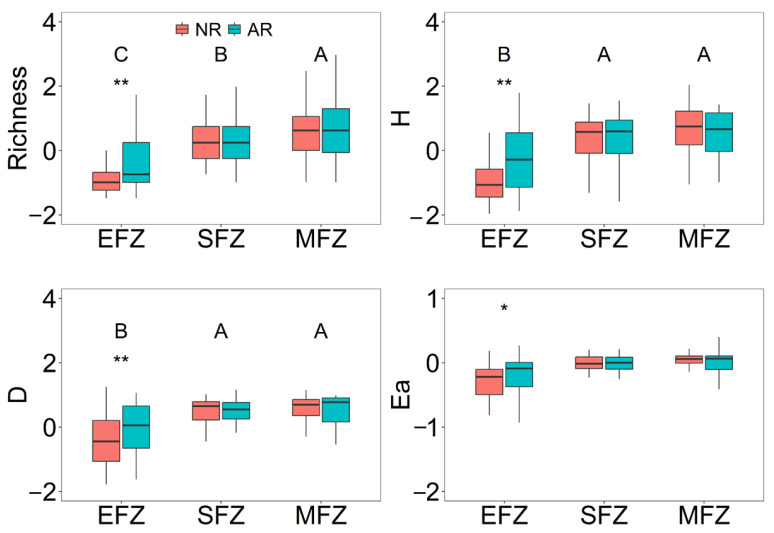
The effects of active revegetation and natural rewilding on the plant diversity indices. “EFZ”, “SFZ”, and “MFZ” represent the extreme flooding zone, severe flooding zone, and moderate flooding zone, respectively. Different letters represent significant differences between flooding zones. “NR” and “AR” represent natural rewilding and active revegetation. “*”, “**” represent the significant differences between natural rewilding and active revegetation at the levels of *p* < 0.05 and *p* < 0.01, respectively.

**Figure 6:**
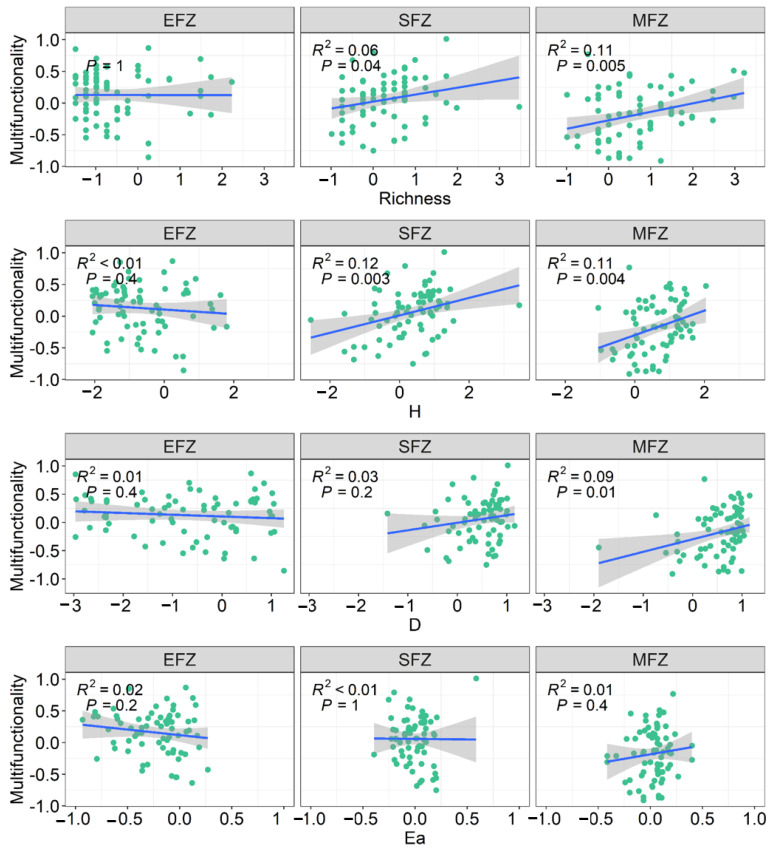
The relationships between plant diversity and ecosystem multifunctionality under different flooding intensities. “EFZ”, “SFZ”, and “MFZ” represent the extreme flooding zone, severe flooding zone, and moderate flooding zone, respectively. The richness, Shannon–Wiener index (H), Simpson’s index (D), and Alatalo’s evenness index (Ea) were the four indices of plant diversity.

Biodiversity was significantly reduced with the flooding intensity, with the lowest biodiversity at the EFZ (Figure 5). Decades of active revegetation increased the biodiversity by 13~57%, which was only observed at the EFZ, compared with natural rewilding. In addition, there was a significant relationship between biodiversity and EMF at the SFZ and MFZ (Figure 6).

**Figure 7:**
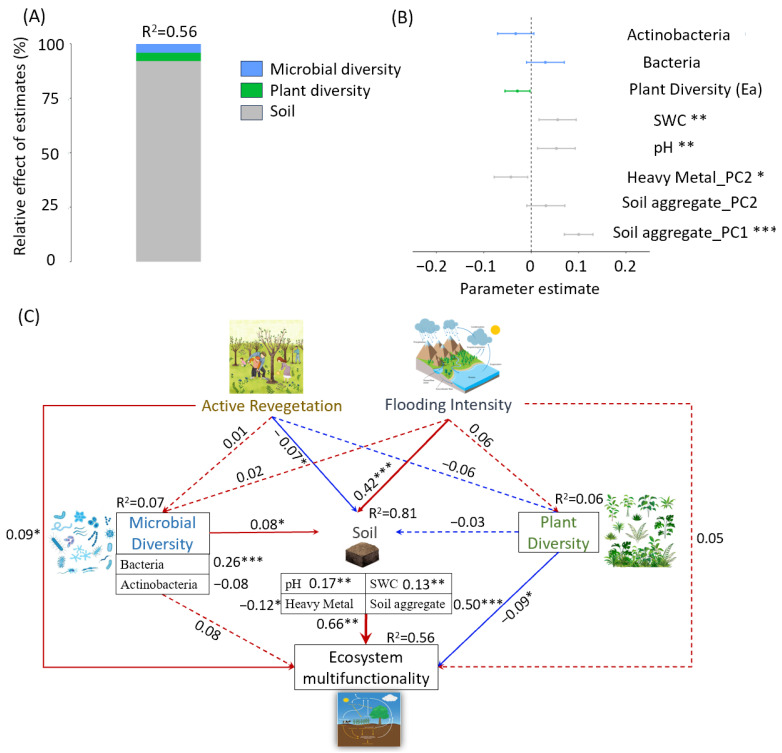
(**A**) The relative importance of the predictors of ecosystem multifunctionality (EMF). (**B**) The coefficients and 95% confidence intervals of each predictor. (**C**) The direct and indirect effects of revegetation and the flooding intensity on EMF (Fisher’s C = 1.64 with *p*-value = 0.44). The red line represents the positive effect, the blue line represents the negative effect, and the dashed line represents insignificant effects. “*”, “**”, and “***” represent significant effects at the levels of 0.05, 0.01, and 0.001, respectively.

According to the multiple regression models, we found that biodiversity and the soil properties were combined to regulate the EMF of the riparian ecosystem (Figure 7A). The soil properties (SWC, pH, soil aggregates, heavy metal content) played a more essential role than biodiversity and explained 54% of the EMF variation (Figure 7B). In addition, active revegetation impacted EMF both directly and indirectly via altering the soil properties (aggregates, heavy metal content, SWC, and pH), while the flooding intensity impacted EMF only indirectly (Figure 7C).

## 4. Discussion

### 4.1. The Effects of Active Revegetation and Natural Rewilding on Ecosystem Multifunctionality

Revegetation is considered an effective restoration strategy in protecting biodiversity, maintaining habitat complexity, and enhancing human well-being [1,2]. Understanding the efficiency of revegetation in restoring multiple functions of riparian ecosystems is critical to develop strategies for the management of global disturbed riparian ecosystems. Here, we investigated the different restoration approaches’ (active revegetation vs. natural rewilding) impacts on the EMF of a riparian ecosystem under contrasting flooding levels. First, we found that active revegetation was more effective than natural rewilding in recovering the EMF at the extreme flooding zone. Second, our system presented important trade-offs between plant biomass, soil fertility, and carbon storage, which need to be considered during restoration processes. Third, we further highlight the role of soil aggregates, the heavy metal content, the pH, and the SWC in supporting EMF in riparian ecosystems. These findings are of paramount importance in guiding the management of riparian ecosystems during restoration efforts.

Riparian ecosystems are subjected to frequent flooding caused by climate change and human activities such as hydroengineering, which significantly reduces biodiversity. For instance, the vegetation diversity decreased by over 70% within the riparian zone of the Three Gorges Dam [18]. We also found that the biodiversity in the riparian ecosystem significantly decreased with the flooding intensity (Figure 5). To recover the biodiversity, revegetation has been widely implemented [27,28]. Previous studies have reported that the plant, animal, and microbial diversity are considerably increased by revegetation [29,30,31]. Indeed, the positive effect of active revegetation on biodiversity was observed at the extreme flooding zone of the riparian ecosystem (Figure 4). As expected, we observed higher EMF at the EFZ of the revegetation site. However, it should be noted that the higher EMF was not due to increasing biodiversity, which had a weak relation with EMF (Figure 6) and did not significantly impact EMF according to the structural equation modeling (Figure 7C). This result is different from the general idea that biodiversity promotes multifunctionality [32], indicating the unique response of riparian ecosystems to revegetation restoration.

Revegetation could impact EMF in both indirect and direct ways at the EFZs of riparian ecosystems. It can alter the soil properties (aggregates, pH, SWC, and heavy metal content) and subsequently change the EMF. This is partly in line with previous studies that reported that the soil properties are equally as important as biodiversity in regulating EMF [13,15,16]. In addition, it directly impacted EMF via positive effects on individual functions. In this study, revegetation increased the plant biomass, soil carbon storage, nutrient cycling, and microbial habitats when compared with natural rewilding (Figure 2), which has also been reported before [4,5,8]. Although these effects were not statistically significant, their combined positive effect would be considerable.

In contrast to the EFZ, there was no substantial difference between active revegetation and natural rewilding in EMF at the SFZ and MFZ of the riparian ecosystem, suggesting that revegetation is equally as effective as natural rewilding in riparian zones with a relatively lower fooding intensity. This result is in line with previous studies demonstrating that natural rewilding is the most cost-effective approach for the recovery of biodiversity, ecological processes, and ecosystem services under favorable ecological conditions [33,34]. Active restoration is often favored in areas where natural rewilding is hindered, such as those experiencing extensive deforestation, intensive disturbances, severe soil degradation, or the loss of the seed bank and root sprouts [33]. It is noteworthy that, in terms of individual functions, revegetation had a negative impact on the soil fertility at the SFZ, which could be attributed to the different species after revegetation. For example, active revegetation uses shrub species such as Morus alba, Hibiscus syriacus, and Salix variegate. However, natural rewilding results in herb species such as *Bidens tripartita, Echinochloa crusgalli*, *C. dactylon*, *Eupatorium adenophorum*, and *Alternanthera philoxeroides* at the SFZ [35]. Compared with herbs, shrub growth needs more nutrients [36], which could explain the reductions in soil fertility after active revegetation. Consequently, for degraded riparian ecosystems that have the potential to be recovered by natural rewilding, active revegetation is not the best candidate to support EMF at the SFZ and MFZ. This study could serve as a fundamental reference in comprehending the potential impact of the flooding intensity on restoration endeavors and offers guidance for the rehabilitation of disrupted riparian ecosystems. However, we do not have information about the situation at the beginning of recovery, and revegetation work is often very important in the early intervention stage; this prevents the comprehensive evaluation of the revegetation effects. Furthermore, under limited resource conditions, recovery to a certain stage may be difficult to continue, or it may have reached a certain recovery threshold. Further research should focus on this aspect and advance our knowledge of the effects of revegetation in riparian ecosystems.

Among the four individual functions, there was a significant trade-off between plant biomass and soil functions (soil carbon storage and fertility), which could be reflected in their negative relationships and opposite responses to the flooding intensity (Figure 2 and Figure 4). Generally, increased plant biomass indicates an increase in photosynthetically derived carbon and greater litter deposition into the soil, improving the soil carbon stock and fertility [5,20]. This unexpected result in this study can be attributed to periodic flooding every year. Flooding could wash away the litter and then reduce the effect of plant productivity on the soil carbon stock and fertility. Moreover, vegetation growth needs more nutrient uptake, thus reducing the soil fertility. Furthermore, the plant biomass and soil functions responded to the flooding intensity in opposite ways (Figure 2). Compared with the SEZ and MFZ, the more intensive flooding at the EFZ significantly increased the soil fertility and soil carbon storage, due to the relatively longer flooding duration and more sediment deposits at the EFZ. This phenomenon has also been observed in coastal ecosystems [37]. However, fewer species can adapt to the intensive flooding at the EFZ; thus, the plant biomass was lower. These findings imply that proactive management measures may be necessary to maintain the functions of plant biomass, soil carbon storage, and fertility simultaneously, such as selecting plants with high nutrient use efficiency, so as to promote long-term sustainable ecosystems.

In addition to the trade-offs, the essential drivers need to be clearly identified so as to successfully restore and sustain EMF. We found positive effects of soil aggregates, the pH, and moisture and negative effects of the heavy metal content on the EMF in the riparian ecosystem. The soil aggregates, functioning as the physical structure of the soil habitat, regulate the resource accessibility of particular microbial populations, consequently shaping interactions among organisms and their associated functions [38,39]. Meanwhile, soil with different particle sizes varies in the quantity and quality of its organic carbon, which is related to soil carbon storage [40]. The soil pH could impact EMF via changing the microbial characteristics and enzyme activity [41], while soil moisture impacts EMF owing to improvements in plant productivity and nutrient availability [42,43,44]. In this study, the high soil aggregate, pH, and moisture levels could promote EMF in the riparian ecosystem (Figure 7). Furthermore, heavy metals exert detrimental impacts on microbes and their functions [45]. To withstand heavy metal stress, microorganisms elevate their energy investments and increase soil carbon release [45]. Therefore, the negative effect of heavy metals was observed. These results suggest that maintaining the soil environment with appropriate pH, aggregate, moisture, and heavy metal content levels is an essential strategy in recovering the EMF of riparian ecosystems.

### 4.2. Implications

Active revegetation requires significant investments in terms of labor, materials, and time. In particular, these are found to be one order of magnitude higher than those for natural rewilding [46]. In addition, active revegetation often needs long-term maintenance to ensure the success of the restoration efforts, including regular monitoring, protection from disturbances, and sometimes additional interventions to support the growth and establishment of the planted species [33]. Although active revegetation costs a lot, it benefits biodiversity and ecosystem services, such as the water supply, soil fertility, and climate regulation [47,48,49]. A previous study has reported that active revegetation generated USD 6.86 billion in the agro-pastoral ecotone of Northern China [50]. However, in terms of riparian ecosystems, we found that active revegetation had a limited effect on multifunctionality, although it significantly increased the biodiversity. This result indicates that natural rewilding is a better way to restore degraded riparian ecosystems. For those sites without the potential for regeneration, active revegetation is a necessity.

## 5. Conclusions

Restoring riparian ecosystems has emerged as a fundamental goal in land management initiatives worldwide. This study provides compelling evidence that a decade of active revegetation is only effective for EMF at the extreme flooding zones of riparian ecosystems. At the severe and moderate flooding zones with a low flooding intensity (below 237 days per year), natural rewilding is an effective and low-cost way to recover EMF. In addition, our study has, for the first time, elucidated a significant trade-off between plant biomass and soil carbon stocks and fertility in riparian ecosystems, which underscores the potential need for management interventions to sustain all these functions. Furthermore, this study sheds light on the role of soil aggregates, pH, moisture, and heavy metal content in riparian ecosystem restoration. By paying more attention to ecosystem restoration to combat biodiversity loss, this research shows that it is important to strengthen the preservation and management of riparian ecosystems under global change. Further research should focus on the effects of natural rewilding and active revegetation on other functions, such as greenhouse gas emissions and the nitrogen removal efficiency, which have not been considered in this study.

## Data Availability

The data are available via Figshare: https://doi.org/10.6084/m9.figshare.23897703.v1.
